# Contact Calls of the Northern and Southern White Rhinoceros Allow for Individual and Species Identification

**DOI:** 10.1371/journal.pone.0098475

**Published:** 2014-06-05

**Authors:** Ivana Cinková, Richard Policht

**Affiliations:** 1 Department of Zoology and Laboratory of Ornithology, Faculty of Science, Palacký University, Olomouc, Czech Republic; 2 Department of Ethology, Institute of Animal Science, Prague, Czech Republic; 3 Department of Game Management and Wildlife Biology, Faculty of Forestry and Wood Sciences, Czech University of Life Sciences Prague, Prague, Czech Republic; University of Saint-Etienne, France

## Abstract

Inter-individual relationships particularly in socially living mammals often require a well-developed communication system. Vocal and olfactory signals are the most important for the communication of rhinos, however, their vocal communication has been investigated to a very limited extent so far. White rhinos have the most developed social system out of all the rhinoceros species and vocal signals might therefore play an important role in their social interactions. We recorded repetitive contact pant calls from six captive northern white rhinos (*Ceratotherium cottoni*) and 14 captive and free-ranging southern white rhinos (*Ceratotherium simum*) and examined if they transmit information about individual identity, species, social context and age class. Discriminant analyses revealed that a high percentage of the pant calls of both species could be classified to a correct individual. We calculated signature information capacity of pant calls recorded from adult animals in isolation at 3.19 bits for the northern white rhinos and at 3.15 bits for the southern white rhinos, which can potentially allow for a vocal discrimination of nine individuals of both species. We found that pant calls varied by species. Northern white rhinos had longer calls and also differed from the southern white rhinos in several frequency parameters of their calls. We also analysed the pant calls of southern white rhinos for the differences between the age classes and between social contexts in which they were recorded. Our results show that pant calls carry information about individual, species, age class and context. The ability to recognize this information would allow rhinos, in addition to olfactory cues, to communicate with highly increased accuracy. A better understanding of communication of white rhinos has potential practical use in their management and conservation particularly because of the low breeding success of white rhinos in captivity.

## Introduction

Certain aspects of rhinoceros acoustic communication have been compared to the communication of elephants intensively studied over recent decades (e.g. [1]–[3]). It has been hypothesised that rhinos might be using infrasound for long-distance communication which would be similar to the communication of elephants [4], [5]. To the best of our knowledge, however, only basic descriptions of the rhinoceros vocal repertoire are known so far and no studies have reported any detailed information encoded in particular rhinoceros calls [6]–[8].

White rhinos have the widest vocal repertoire out of all the rhinoceros species whose vocalizations have been studied bioacoustically (see [6]–[8]) and also have the most developed social system (see [9]–[13]). Adult white rhinoceros males are territorial while females, subadults and juveniles live in groups in overlapping home-ranges [10], [11], [14]. The most frequently observed long-term associations of southern white rhinos include 2–3 individuals, although long-lasting groupings of up to six animals have also been recorded [10], [15]. Advanced acoustic communication might therefore be particularly useful for the white rhinoceros. Vocal recognition of offspring [16], [17], mother [18], sex or age class [19], [20], group membership [21], [22], individual identity [23], [24] and the dominant or subordinate status of males [25] have been previously described in many socially living mammals.

Acoustic signals may also serve as a premating isolating mechanism and restrict reproduction between different species [26]. Two subspecies of the white rhinoceros have recently been elevated to the species level, the northern (*Ceratotherium cottoni*) and southern white rhinoceros (*Ceratotherium simum*) [27]. More detailed research on their possible vocal distinction might contribute valuable data to this reassessment. Rookmaaker [28], [29] has suggested that due to taxonomic revision, the name northern white rhinoceros is no longer appropriate and suggests following Heller [30] and calling it the Nile rhinoceros.

Rhinos are known to utter calls belonging to several categories including puffing, growling and harmonic calls (see [6]–[8], [10], [12]). The vocal repertoire of black [6], Sumatran [7] and northern white rhinos [8] has been studied bioacoustically while the repertoire of Indian [12] and southern white rhinos [10] has only been described verbally. Northern and southern white rhinos share a similar vocal repertoire [8], [10] and a detailed comparison of the vocal repertoires of all rhinoceros species revealed that white rhinos use a unique category of repetitive calls [8].

Policht et al. [8] suggested that the repetitive contact call pant of white rhinos, which is not known in other rhinoceros species, could be used for long-distance communication. White rhinos live in open habitats [10], [31] and the repetition of short signals would therefore be favoured for long-distance communication in this environment due to an easier detection between bursts of wind [32]. Pant is a sequence of inhalations and exhalations and is used by all sex-age classes when greeting or approaching another rhinoceros, as a response to previous calls or during separation from a group [8], [10]. Pant is usually apparently directed to a particular individual [8] and in such vocalizations, individuality in calls could be expected. The differences in vocalizations between males can also indicate the quality (red deer: [33]) or social status (horse: [22]) of the male and therefore influence female mate choice. Vocal individuality also has the potential to be used in conservation; individual discrimination and identification of animals have their implications from census tasks to monitoring the animals over time [34].

Northern white rhinos are currently on the brink of extinction with only seven surviving individuals and although the numbers of southern white rhinos have recently reached over 20,000 individuals in Africa, their population is in danger due to escalating poaching [35]. The reproduction of both species in captivity is extremely low (e.g. [36], [37]). Although the reasons behind this are poorly understood, several studies have suggested that social interactions between captive rhinos might be one of the possible reasons [38]–[41]. Research on white rhinoceros communication might be extremely valuable for an improved understanding of their social behaviour.

We investigated whether the contact pant calls of white rhinos contain sufficient information for recognition of individuals, species, age classes and contexts. In addition, we also calculated the signature information capacity H_S_ present in the pant calls of northern and southern white rhinos following Beecher [42].

## Materials and Methods

### Ethics Statement

Research for this project including the recording of calls and playbacks of pant calls for white rhinos was approved by the Ethics and Scientific Committee of the National Zoological Gardens of South Africa (Project P11/03). The research was conducted in accordance with the guidelines of the Animal Behaviour Society for the ethical use of animals in research.

### Animals and Data Collection

The pant calls of six northern white rhinos were recorded in the zoological garden Dvůr Králové and the calls of 14 southern white rhinos in zoological gardens Salzburg, Zlín, Bratislava, Dvůr Králové and in the South African wildlife reserves Lapalala Wilderness, Welgevonden Game Reserve and Lichtenburg Biodiversity Conservation Centre in 2005–2006 and 2009–2012 (Table 1). We recorded the calls with a Sennheiser directional microphone (ME 67 with K6 powering module, frequency response: 40–20,000 Hz±2.5 db) fitted with a Rycote Softie windshield and digital recorders Marantz PMD 671 or Yamaha Pocketrak C24 with a 44.1 kHz sampling rate and 16 bits resolution. The vocalizations were recorded both outside and in the stables at distances from 0.5 to 30 m, over a minimum of two different days for each animal (mean 5±2.5 days, range 2–12 days) and with the time between the first and last recording varying from three days to five years for each subject.

**Table 1:** Characteristics of the animals included in the study.

Individual (studbook no.)	Sex	Age (years)^1^	Population	Zoo/Reserve	No. of calls analysed in each context^2^				Notes
					Total	I	PI	VC	
**Northern white rhinos:**									
Fatu (#1305)	F	S, A (5,6,9)	Zoo	Dvůr Králové	35	26	5	4	Parents Nájin x Saút
Nabiré (#0789)	F	A (25,26)	Zoo	Dvůr Králové	53	24	18	11	Parents Nasima x Súdán
Nájin (#0943)	F	A (17,20)	Zoo	Dvůr Králové	34	24	10	–	Parents Nasima x Súdán
Nesárí (#0377)	F	A (33,37,38)	Zoo	Dvůr Králové	15	6	5	4	Wild-born
Súdán (#0372)	M	A (36)	Zoo	Dvůr Králové	18	18	–	–	Wild-born
Suni (#0630)	M	A (29)	Zoo	Dvůr Králové	8	8	–	–	Parents Nasima x Saút
**Southern white rhinos:**									
Ada (#1154)	F	A (25)	Zoo	Bratislava	12	12	–	–	Wild-born
Kathi (#362)	F	A (37,38)	Zoo	Salzburg	23	16	–	7	Wild-born
Kifaru (#773)	F	A (27)	Zoo	Salzburg	21	21	–	–	
Munyani	F	A (15)	Free-ranging	Lapalala	35	35	–	–	Wild-born
Yeti (#936)	F	A (11)	Zoo	Salzburg	9	–	–	9	Wild-born, mother of Malia
Malia (#938)	F	S (3)	Zoo	Salzburg	7	–	–	7	Wild-born, daughter of Yeti
Tamu (#937)	F	S (4)	Zoo	Salzburg	19	–	–	19	Wild-born
Naja (#T18)	F	S (5)	Zoo	Zlín	17	13	–	4	Wild-born
Zanta (#T17)	F	S (6)	Zoo	Zlín	26	24	–	2	Wild-born
Natal (#371)	M	A (39)	Zoo	Dvůr Králové	25	25	–	–	Wild-born
Medupi	M	S (3)	Free-ranging	Lapalala	10	10	–	–	Wild-born, stepbrother of Lekoto
Lekoto	M	S (2)	Free-ranging	Lapalala	5	–	–	5	Wild-born, stepbrother of Medupi
M06	M	S (4)	Free-ranging	Welgevonden	8	–	–	8	Wild-born
Bert	M	S (3)	Free-ranging	Lichtenburg	5	–	1	4	Wild-born

1 Age at time of call recordings. A = adult, S = subadult. The animals were classified by age class following Owen-Smith [10], [11]; females were regarded as adults at 6.5–7 years of age and males between 10 and 12 years of age.

2 I = visual isolation from other rhinos, PI = partial isolation: the animal was visually isolated from the rest of its group, but was in the company of other rhinos, VC = in visual contact with group member(s).

The pant calls were recorded in the following context: (1) visual isolation from other white rhinos (68.1% of the calls), (2) partial isolation: the animal was visually isolated from the rest of its group, but was in the company of other white rhinos (10.1%), (3) in visual contact with group member(s) (21.8%). The animals vocalized either naturally or replied to our playback of a pant call. The rhinos became isolated when they either naturally separated themselves and lost visual contact or when they were separated in the enclosure or in the stables by the keepers. All the pant calls of adult males were recorded in visual isolation from other white rhinos.

### Acoustic Analysis

We only included complete calls consisting of a series of elements in the analysis; single inhalations or exhalations, which rhinos occasionally produce in excited situations, were not analysed. We only selected calls which were recorded in good quality with low background noise. The call elements were manually marked in Avisoft SAS Lab Pro 5.2.07 (Avisoft Bioacoustics, R. Specht, Berlin, Germany) with the help of an envelope curve and spectrogram. The temporal parameters were then computed automatically using the following spectrogram parameters: FFT length 256, frame size 100%, overlap 50%, FlatTop window. These were duration, interval between particular elements, the distance from the start to maximum amplitude and start/end time. As the duration of the particular elements and the number of elements of a certain duration within the call were highly variable between individuals, we calculated various parameters in order to extract the most important temporal characteristics of the calls. We calculated the number of elements in each call, the call duration, the duration of the longest and shortest inhalation and exhalation in the call, the order of the longest inhalation and exhalation in the call, the number of inhalations and exhalations in the call in the duration from 0.0–0.4 sec ( = in categories 1 and 2, see below) and the percentage of inhalations and exhalations in a duration 0.0–0.4 sec from all the inhalations and exhalations in the call. Spectral parameters were measured using the following spectrogram parameters: FFT length 1,024, frame size 100%, overlap 87.5%, Hamming window.

**Figure 1:**
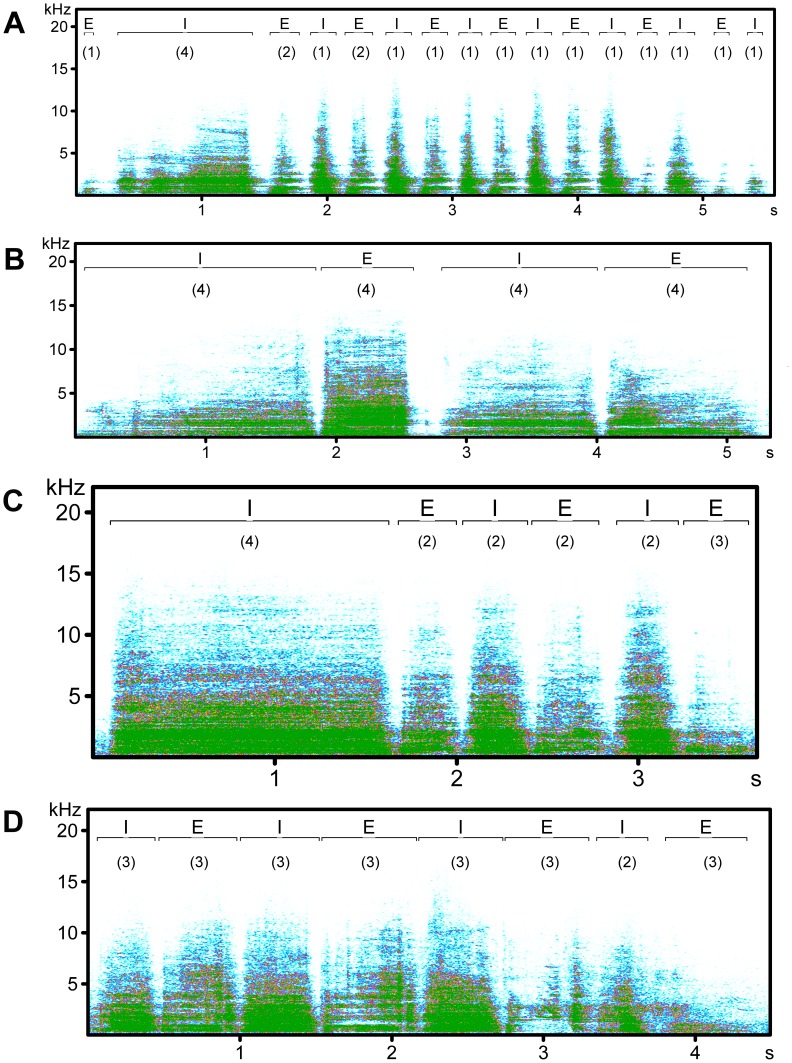
Spectrograms of pant calls of adult northern and southern white rhinos. Northern white rhinos: female Nabiré (A) and male Suni (B). Southern white rhinos: female Yeti (C) and male Natal (D). Inhalations (I), exhalations (E) and their affiliation to the categories based on their duration are shown: category (1): 0.0–0.2 sec, (2): 0.21–0.4 sec, (3): 0.41–0.8 sec, (4): 0.81 sec. (Spectrogram parameters: FFT length 1024, frame size 100%, overlap 87.5%, Hamming window).

As pant calls are repetitive signals, we only selected certain elements of the calls to measure the spectral parameters. The calls were divided into inhalations and exhalations and several very weak elements (2% out of the total number of elements) were excluded from the analysis to avoid fluctuations in breath intensity. The inhalations and exhalations were then divided according to their duration into categories: (1) 0.0–0.2 sec (28.6% of all elements), (2) 0.21–0.4 sec (50.1%), (3) 0.41–0.8 sec (18.6%), (4) >0.81 sec (2.7%) (Figure 1). Only one element from the most numerous group of inhalations and exhalations was chosen for the analysis (in 2.3% of inhalations and 3.6% of exhalations, we used an element from the second most numerous group as there was no good quality recorded element in the first group). Within the group, the first well-recorded intensive element from the beginning of each call was chosen. The spectrograms (spectrogram parameters: FFT length 1024, frame size 100%, overlap 87.5%, Hamming window, time resolution 2.9 ms) of these elements were then analysed in the acoustic programme LMA 2008 (kindly provided by Kurt Hammerschmidt) and we computed 117 parameters for each selected element.

### General Statistical Analyses

All the variables were Box-Cox transformed to improve the normality of their distribution. The dataset contained a few zero values; they were therefore shifted to the smallest possible value during the Box-Cox transformation. Statistical analyses were performed with software IBM SPSS Statistics 20.0 (IBM Corp., Armonk, USA) (for conventional discriminant function analyses and information calculation), R 3.0.2 (R Foundation for Statistical Computing, Vienna, Austria) (for permuted discriminant function analyses) and Statistica 12.0 (StatSoft Inc., Tulsa, USA) (for Box-Cox transformations and Mann-Whitney U tests).

### Discriminant Function Analyses

We performed conventional discriminant function analyses (DFAs) and permuted discriminant function analyses (pDFAs) to test our ability to correctly assign calls to individual, species, context and age class. We checked for pair-wise correlations between variables and only one from a highly correlated pair with r>0.8 has been retained in the analyses.

We conducted conventional forward stepwise DFAs to classify the calls of both species according to the individuals. The variables were added and removed based on the changes in Wilks’ lambda. The resulting variables which contributed to the greatest extent to the recognition between individuals were used as a source for the final DFAs. We applied a cross-validation (leave-one-out procedure) to validate the results of the DFAs as in this case each call in the analysis was classified by the functions derived from all the calls other than that call. We studied the possibility of classifying the calls of northern white rhinos recorded in various social contexts to a correct individual ( = DFA 1). To avoid overestimation or underestimation of our results due to the fact that the animals were recorded in various contexts, we re-ran the analysis using only calls recorded in isolation ( = DFA 2). Similarly, we performed DFA 3 to study the possibility of correctly classifying the calls of southern white rhinos recorded in various contexts as well as calls recorded only in isolation ( = DFA 4). The DFAs 2 and 4 were only conducted using the calls of the adult animals to control for any possible influence of age class on our results.

We then performed pDFAs for nested designs, which is a randomization procedure used for non-independent two-factorial data sets when one factor is nested in another. The detailed procedure is described in Mundry and Sommer [43]. The pDFAs were conducted using a script written in software R (kindly provided by Roger Mundry) using 100 random selections and 1,000 permutations. The script is based on the function Ida of the R package MASS [44]. The pDFA calculates the percentage of correctly classified objects for the original (i.e. unpermuted) data, based on the calls used to derive discriminant functions and the percentage of correctly classified calls for the cross-validated (i.e. permuted) data, which were not used to derive discriminant functions [43].

We conducted pDFAs to test our ability to correctly assign calls to context ( = pDFA 1) and age class (adults and subadults;  = DFA 2) in southern white rhinos and to assign calls of adult northern and southern white rhinos recorded in isolation to correct species ( = pDFA 3) while controlling for individual variation. For pDFAs, we used variables which discriminated best between contexts and age classes in southern white rhinos and between species. In pDFA 1, we included calls from animals recorded in isolation (Ada, Kathi, Kifaru, Munyani, Naja, Zanta, Natal, Medupi) and in visual contact with group member(s) (Yeti, Malia, Tamu, Lekoto, M06, Bert) in order to meet the requirements of the nested design of pDFA as the calls of each individual can only be included in one context. The same analysis was not performed for the northern white rhinos as we were limited by the number of individuals and the number of recorded calls in each context.

We conducted seven DFAs and pDFAs in total. A sequential Bonferroni correction was therefore applied to correct the p-values. A detailed description of all the variables used in the DFAs is provided in Table S1 and their descriptive statistics in Table S2. Two-tailed Mann-Whitney U tests were performed to test for differences in the call parameters between species and between contexts and age classes in southern white rhinos. We applied the sequential Bonferroni correction as the data were used for multiple comparisons.

### Information Calculation

We described the variability in calls of northern and southern white rhinos following Beecher [42], Arnold and Wilkinson [45] and Carter et al. [46]. We extracted principal components (PCs) with varimax rotation from call data and ran a parallel analysis [47] to determine how many PCs to extract from our data. We saved the PC scores using the Bartlett method and then used the restricted maximum likelihood to obtain the variance component estimate (VCE) of the random factors (individual, sex, context, age class, population) for each retained PC. We weighted the VCE for each factor by the percentage variance explained by its corresponding PC to estimate the percentage of variance contributed by the random factors. As the southern white rhinos were recorded both in zoological gardens and in wildlife reserves, we divided them into two groups according to the population (captive and free-ranging) and included population as a random factor in the VCE analysis.

The signature information capacity in contact calls was calculated following Beecher [42], Arnold and Wilkinson [45] and Carter et al. [46]. We favoured this approach as it allows for a comparison of signature information content in calls across different species or sample sizes [42] and it is a standard method used in many recent papers (e.g. [45], [46], [48]–[51]). The information capacity H_S_ in a particular vocalization is calculated in bits [42]. The value 2^HS^ provides an estimate of the number of individuals, which can potentially be discriminated on the basis of the call parameters considered [52]. To avoid any influence of call context or age class on our results, we calculated H_S_ from the calls of adult northern and southern white rhinos recorded in isolation. The total signature information capacity was calculated from VCEs (obtained by the procedure described above) for individual and sex differences (S_B_^2^) and within-individual differences ( = unexplained variation in calls) (S_W_^2^). The total variance (S_T_^2^) is the sum of S_B_^2^ and S_W_^2^. The information in each PC was then summed (H_i_ = log_2_ (S_T_/S_W_)) to calculate the total information capacity in the call (H_S_ = ∑H_i_) and the repeatability of each PC (S_B_^2^/(S_B_^2^+S_W_^2^)) [42], [45], [46].

## Results

### Description of the Pant Calls of Both Species

We recorded 163 calls of six northern white rhinos, which consisted on average of 14±4.7 elements with a call duration 6±1.8 sec. The mean frequency range was 4794±1609.6 Hz in inhalation and 4948±2119.1 Hz in exhalation. The minimum frequency of the first and maximum frequency of the third distribution of the frequency amplitude was 175±139.8 Hz and 9170±3870.6 Hz, respectively in inhalation and 106±102.1 Hz and 8351±3825.6 Hz, respectively in exhalation. We recorded 222 calls of 14 southern white rhinos, which consisted on average of 9±2.6 elements with a call duration 4±0.9 sec. The mean frequency range was 4504±1362.6 Hz in inhalation and 5753±1678.2 Hz in exhalation. The minimum frequency of the first and maximum frequency of the third distribution of the frequency amplitude was 530±260.2 Hz and 12003±4269.8 Hz, respectively in inhalation and 422±206.2 Hz and 14768±2757.1 Hz, respectively in exhalation (see Figure 1).

### Information Encoded in the Calls of Northern White Rhinos

**Figure 2:**
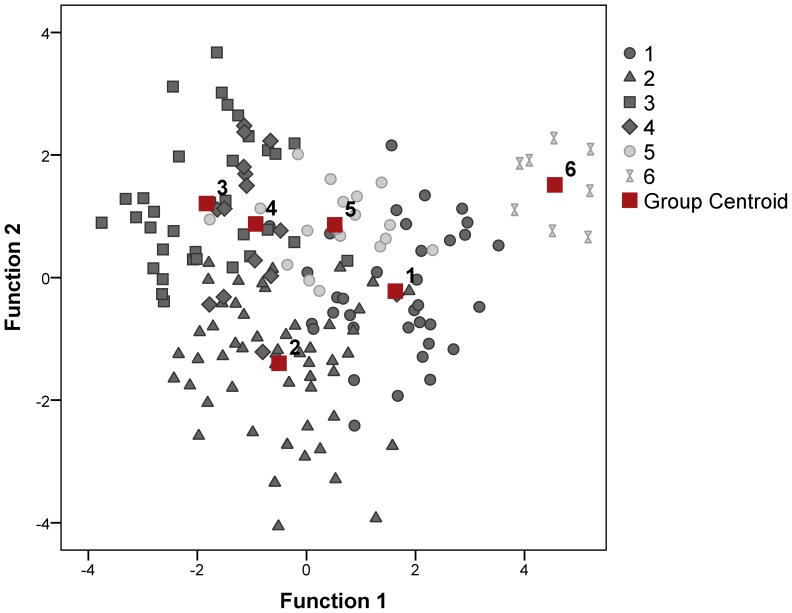
Vocal individuality in pant calls of northern white rhinos recorded in various social contexts. The plot shows the first two canonical discriminant functions with the centroid values of pant calls for each animal. 1 = Fatu, 2 = Nabiré, 3 = Nájin, 4 = Nesárí, 5 = Súdán, 6 = Suni.

The pant calls of northern white rhinos were individually distinct. A discriminant function analysis assigned 81% of calls (74% cross-validated) to the correct individual (DFA 1: N = 6 animals, n = 163 calls, Wilks’ lambda = 0.064, p<0.001) (Table 2, Figure 2). We included nine acoustic variables into this model (Table 3). Three extracted principal components from this model accounted for 57% of the total variance in calls. Out of this variation, 32.4% was explained by individual (14.5%), sex (10.8%), context (1.5%), interaction between the individual and context (4.7%) and between the sex and context (0.9%).

**Table 2:** Classification success of pant calls of northern and southern white rhinos in conventional discriminant function analyses conducted with calls recorded from all the animals in various contexts (DFAs 1, 3) and with calls recorded only from the adult animals in isolation (DFAs 2, 4).

Individual	Sex	Calls recorded in various contexts		Calls recorded in isolation	
		% correctly classified	% correctly classifiedcross-validated	% correctlyclassified	% correctly classifiedcross-validated
**Northern white rhinos:**					
		DFA 1	DFA 1	DFA 2	DFA 2
Fatu	F	85.7	68.6	83.3	79.2
Nabiré	F	75.5	75.5	75.0	70.8
Nájin	F	79.4	76.5	87.5	75.0
Nesárí	F	86.7	66.7	100	83.3
Súdán	M	77.8	72.2	94.4	83.3
Suni	M	100	100	100	100
**Southern white rhinos:**					
		DFA 3	DFA 3	DFA 4	DFA 4
Ada	F	100	91.7	100	83.3
Kathi	F	95.7	95.7	93.8	81.3
Kifaru	F	85.7	71.4	85.7	81.0
Munyani	F	97.1	88.6	80.0	80.0
Yeti	F	88.9	55.6	–	–
Malia	F	100	71.4	–	–
Tamu	F	68.4	63.2	–	–
Naja	F	94.1	88.2	–	–
Zanta	F	96.2	84.6	–	–
Natal	M	100	96.0	100	92.0
Medupi	M	100	90.0	–	–
Lekoto	M	60.0	60.0	–	–
M06	M	100	100	–	–
Bert	M	100	60.0	–	–

**Table 3:** DFA structure matrices for northern and southern white rhinos showing pooled within group correlations between discriminating variables and standardized canonical discriminant functions with Eigenvalues>1.

Acoustic variable	Short description	Northern white rhinos		Southern white rhinos				
		DFA 1: Discriminant functions		DFA 3: Discriminant functions				
		1.	2.	1.	2.	3.	4.	5.
Call duration	Duration of call (sec)			−0.41			0.41	
No. elements	Number of elements in call			−0.41			0.47	
I: max element length	Duration of the longest inhalation in call (sec)	0.49						
I: min element length	Duration of the shortest inhalation in call (sec)	0.46	0.38			−0.49		
Order longest inhalation	Order of the longest inhalation in call							0.40
I: no. in cat. 1,2–percentage	Percentage of exhalations in call, which are in duration from 0.0–0.4 sec					0.55		
E: max element length	Duration of the longest exhalation in call (sec)	0.50			0.55			
E: duration	Duration of exhalation (sec)				0.61			−0.38
E: no. in cat.1,2	No. of exhalations in call in duration from 0.0–0.4 sec ( = in categories 1, 2)	−0.45						
I: peak freq (max)	Frequency of the maximum amplitude of spectrum [Hz]		−0.38					
I: ampratio3	Amplitude ratio between 2^nd^ and 3^rd^ dominant frequency band [Hz]		0.36					
I: df3mean	Mean frequency of 3^rd^ dominant frequency band [Hz]		0.35					
Eigenvalue		2.58	1.26	4.01	3.3	2.3	1.6	1.1
% of variance		55.8	27.3	27.4	22.1	15.7	11.0	7.4
Cumulative %		55.8	83.1	27.4	49.6	65.3	76.3	83.7

Only correlations≥0.35 are shown. Eigenvalue and percentage of variance explained by each discriminant function are also shown. The DFA 1 included calls of all the northern white rhinos recorded in various contexts, DFA 3 included calls of all the southern white rhinos recorded in various contexts. I = variable measured in inhalation, E = variable measured in exhalation.

The ability to assign calls to individuals slightly increased when we only included calls recorded from the adult animals in isolation and 87% of calls (79% cross-validated) were correctly classified (DFA 2: N = 6 animals, n = 104 calls, Wilks’ lambda = 0.023, p<0.001) (Table 2). We used the same nine acoustic variables as in DFA 1. The first three discriminant functions with Eigenvalue>1 explained 94% of the variability. Based on this model, we calculated the signature information in the pant calls, which was 3.19 bits with a mean repeatability of PCs 0.52.

### Information Encoded in the Calls of Southern White Rhinos

**Figure 3:**
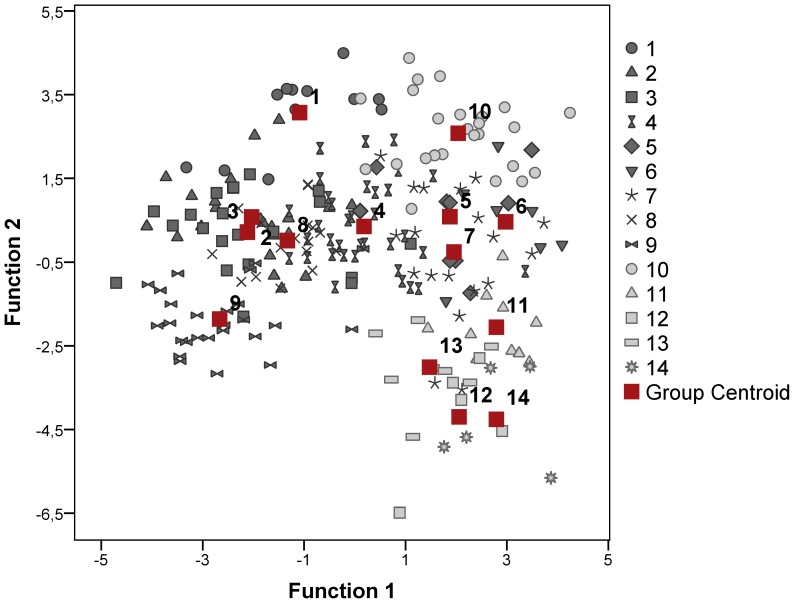
Vocal individuality in pant calls of southern white rhinos recorded in various social contexts. The plot shows the first two canonical discriminant functions with the centroid values of pant calls for each animal. 1 = Ada, 2 = Kathi, 3 = Kifaru, 4 = Munyani, 5 = Yeti, 6 = Malia, 7 = Tamu, 8 = Naja, 9 = Zanta, 10 = Natal, 11 = Medupi, 12 = Lekoto, 13 = M06, 14 = Bert.

The pant calls of southern white rhinos were classified to the correct individual with 93% success (83% cross-validated) (DFA 3: N = 14 animals, n = 222 calls, Wilks’ lambda = 0.0004, p<0.001) (Table 2) by using 17 acoustic variables (Table 3, Figure 3). Six extracted principal components from this model accounted for 73% of the total variance in calls. Out of this variation, 38.8% was explained by individual (9.3%), age class (7.1%), sex (5.0%), population (3.4%), context (0.6%), interaction between the individual and context (6.0%), sex and age class (2.8%), context and age class (1.9%) and further 2.7% of the variation was explained by other interactions between these factors.

As the calls of some animals were primarily recorded in isolation and the calls of others primarily in visual contact with group member(s) (see Table 1), we conducted pDFA to test whether the calls vary by these contexts while controlling for individual variation. By using six variables (Table 4), we could correctly assign 92% of calls (89% cross-validated) (pDFA 1: N = 14 animals, n = 208 calls, p = 0.01). Pant calls of the southern white rhinos also varied by age class. We classified 88% of calls (86% cross-validated) to the correct age class when controlling for individual variation (pDFA 2: N = 14 animals, n = 222 calls, p = 0.028). We included five variables in this model (Table 4).

**Table 4:** Differences in the acoustic parameters of pant calls of the southern white rhinos between the social contexts (pDFA 1) and age classes (pDFA 2).

Acoustic variable^1^	Short description	Mean±sd^2^		p-value^3^
pDFA1		In isolation	In visual contact	
No. of elements	Number of elements in call	10.6±2.58	7.6±2.76	0.07
I: fp1amax	Maximum amplitude of 1^st^ global frequency peak (relative amplitude)	637.2±403.48	276.3±218.61	0.20
I: peak freq (max)	Frequency of maximum amplitude of spectrum [Hz]	1107.6±369.36	1713.5±482.16	0.10
I: q3med	Median frequency of 3^rd^ distribution of frequency amplitude [Hz]	3188.7±894.02	4785.6±1384.89	0.14
E: q3med	Median frequency of 3^rd^ distribution of frequency amplitude [Hz]	3852.5±1075.60	7463.2±1719.25	0.049
E: df1end	End frequency of 1^st^ dominant frequency band [Hz]	771.93±472.60	583.2±500.38	0.27
**pDFA2**		**Adults**	**Subadults**	
Call duration	Duration of the call [sec]	4.6±0.90	3.4±0.98	0.23
E: max element length	Duration of the longest exhalation in call [sec]	0.6±0.14	0.4±0.11	0.08
E: min element length	Duration of the shortest exhalation in call [sec]	0.3±0.10	0.2±0.06	0.23
E: fp1amean	Mean amplitude of the 1^st^ global frequency peak	135.4±110.06	34.1±37.71	0.04
I: ampratio1	Amplitude ratio between 1^st^ and 2^nd^ dominant frequency band	1.3±0.55	0.8±0.25	0.01

1 I = variable measured in inhalation, E = variable measured in exhalation.

2 The data were calculated as averages of mean values/individual.

3 Significance of Mann-Whitney U tests after sequential Bonferroni correction.

We ran another conventional DFA with the calls of adult southern white rhinos recorded in isolation. This analysis revealed similar results as the DFA 3 and 90% of calls (84% cross-validated) were classified to the correct individual (DFA 4: N = 5 animals, n = 109 calls, Wilks’ lambda = 0.028, p<0.001) (Table 2). We included eight variables in this model (these variables were also included in DFA 3) and the first two discriminant functions with Eigenvalue>1 explained 84% of the variability. Based on this model, we calculated signature information in the pant calls, which was 3.15 bits with a mean repeatability of PCs 0.71.

### Species Differences in the Pant Calls

**Figure 4:**
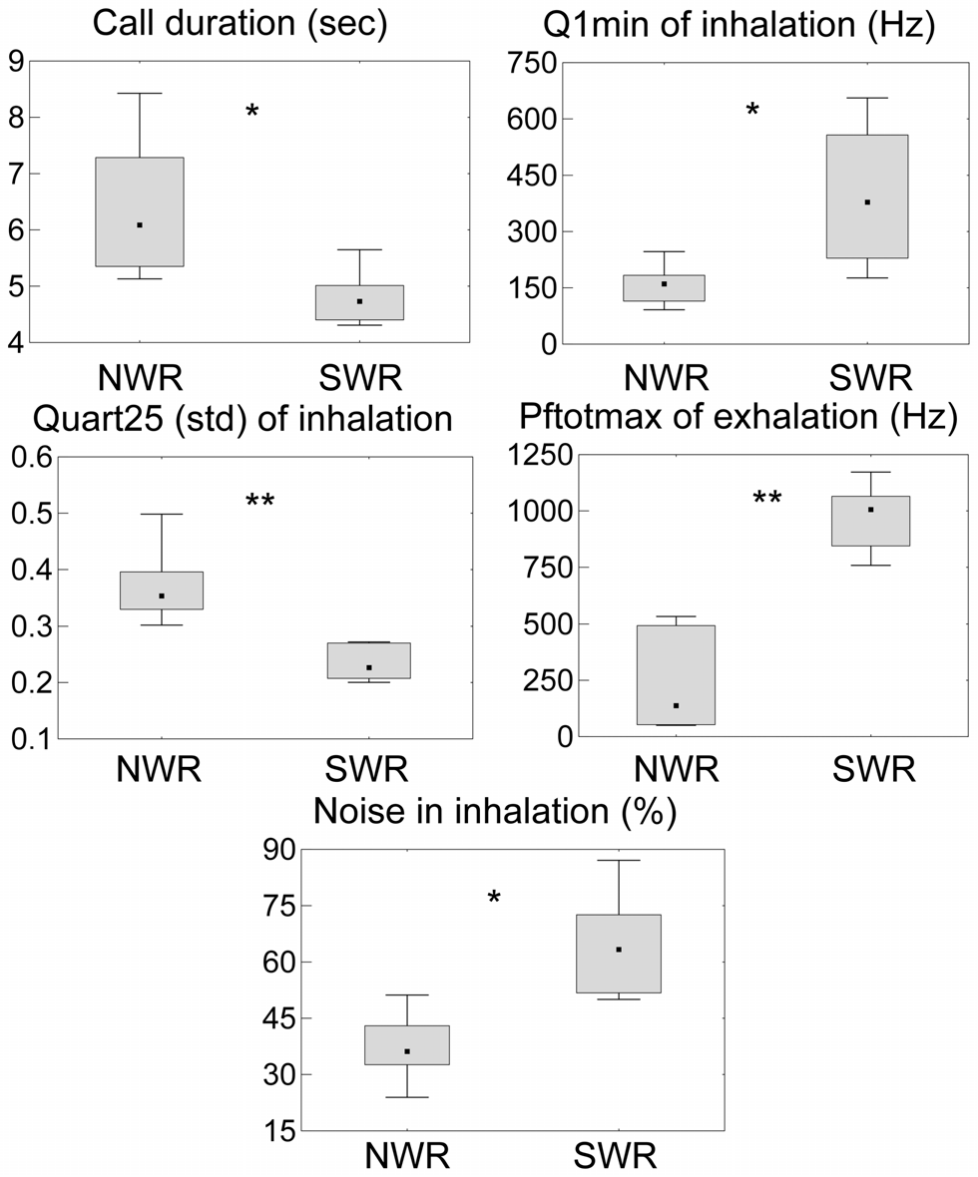
Differences in acoustic variables between the northern (NWR) and southern (SWR) white rhinos. The analysis only included calls of adult animals recorded in isolation. Median, box: 25–75% quartile±maximum, minimum value. Q1min = minimum frequency of the 1^st^ distribution of the frequency amplitude, Pftotmax = frequency of the total maximum amplitude, Quart25 (std) = relative standard deviation of 25% quartile measured from all spectra between the start and the end of the element, Noise = percentage of noisy time segments. Results of Mann-Whitney U tests after sequential Bonferroni correction: *p = 0.07, **p = 0.04.

We conducted pDFA to assess differences in pant calls between the northern and southern white rhinos while controlling for individual variation. To avoid any possible influence of age class or social context on our results, we only included calls from the adult animals recorded in isolation. Five variables were included in this model (Figure 4) and 91% of calls (90% cross-validated) were assigned to the correct species (pDFA 3: N = 11 animals, n = 213 calls, p = 0.01).

## Discussion

### The Function of Pant Call and Factors Influencing its Structure

Despite the growing number of papers devoted to the information encoded in animal vocalizations, such studies have been completely lacking for the entire family *Rhinocerotidae*. Policht et al. [8] suggested that contact call pant of the northern and southern white rhinos might serve for long-distance communication. Since we found that pant carries the individual characteristics of the caller and contains context-, age class- and species-specific acoustic features, we agree that it could serve as a signal advertising the identity and state of the caller at longer distances than can be achieved by visual or olfactory cues, particularly since the eyesight of rhinos is weak (e.g. [31]). Certain parameters of mammalian vocalizations can be highly correlated between relatives [53], however, relatedness does not necessarily affect the overall acoustic similarity between the animals [53], [54]. Although most northern white rhinos in our study were closely related, it seems unlikely that it would affect our results because the calls of unrelated individuals (SÚDÁN and NESÁRÍ) clustered in DFAs 1 and 2 together and also along with other northern white rhinos. Only two pairs of the southern white rhinos were related (see Table 1).

We found that pant calls of the southern white rhinos varied by age class and social context in which they were recorded. Although the differences were not statistically significant, subadults produced calls with shorter total duration and with shorter duration of the longest and shortest exhalation than adults. This is in accordance with our expectations since the smaller animals have smaller lungs and less air volume available for calling. They should therefore emit shorter calls than the larger animals [55]. The five parameters, which we used to classify the calls according to the context also included a number of elements in the calls. The calls emitted in isolation contained more elements than the calls emitted in visual contact with group member(s). More frequent repetition of elements in the pant call could serve to better locate an isolated individual by the group member(s). In king penguins, more frequent repetition of syllables helps the chicks to better locate their parents because of the background noise of the colony [56]. However, when we studied the differences between the age classes and social contexts in the southern white rhinos, both age class categories (adults and subadults) included calls recoded in various social contexts. Similarly, the tested categories of the social context included calls recorded from both the adult and subadult animals (see Table 1). Consequently, we cannot exclude that the differences between the social contexts were not influenced by the differences between the age classes and the other way around. Further studies are therefore necessary to confirm our results.

Pant calls of all four subadult southern white rhinoceros males clustered together in DFA 3 and the calls of adult northern white rhinoceros male SUNI were clearly separated in DFAs 1 and 2 from the calls of other rhinos. This could indicate the influence of age class and social status on the call structure of males. The bull SUNI was kept in the stables with females and with an adult male SÚDÁN, whose calls clustered together with the calls of the females. We think that SUNI might have had a subordinate status as he vocalized with the pant call and answered the playbacks of pant only in the enclosure, where he and not SÚDÁN had access. Unlike SÚDÁN, SUNI was not observed to vocalize with pant call in the stables or in the enclosure, where both bulls had access in turns and where they used olfactory territorial markings. The coding of male social status has also been recorded in squeals [57] and contact whinny calls [22] of horses.

### Species Differences in White Rhinoceros Vocalizations

Signals evolve in correlation with sensory systems, signalling behaviour and micro-habitat choice [58]. Policht et al. [59] found the link between the acoustic parameters of long-range calls and social system in equids. The northern and southern white rhinos have a similar social system [10], [14], [38], [41], [60], however, the northern males produce a unique repetitive call hoarse. All three adult northern white rhinoceros males studied by Policht et al. [8] (two of them were also included in our study) produced hoarse calls primarily during non-social activities such as feeding with its mean duration being 26 sec. Both northern males in our study also vocalized with a hoarse call during almost all feeding bouts. We did not record a hoarse call, however, in any of the three studied captive adult southern white rhinoceros males (two of them were not included in the results due to the low number of recorded pant calls from them) or in the adult males (n = 4) observed during feeding in several zoos, which we visited. This corresponds with the observations concerning the free-ranging southern white rhinos; no hoarse call was recorded during the studies of 11 adult males by Cinková et al. [61] and Cinková and Policht (unpublished data) or during the long-term behavioural study by Owen-Smith [10]. A hoarse call could therefore be considered an apomorphy of the northern white rhinoceros, although, its function remains unclear. This question could be tested by playbacks of this call to both species, but unfortunately only three northern males are currently known to survive.

Species distinction in white rhinoceros puffing and growling sounds was not found by Policht et al. [8], however, their study only included three southern white rhinos, which prevented them from performing a more detailed comparison. We found that the pant calls of northern and southern white rhinos could be classified to a correct species with a high accuracy. This may be the result of the repetitive character and more complex structure of pant calls in contrast to the puffing or growling sounds.

The sensory drive hypothesis for divergence in sexual signalling between closely related species emphasises the adaptation of communication systems to local environments [62]. The southern white rhinos in Kruger National Park in South Africa, where their largest population is found [63], prefer a habitat with good quality short grasses and an open to moderate low shrub stratum in woodland or tree and bush savannah not far from a water source [64], [65]. The northern white rhinos are most probably extinct in the wild [66], however, they formerly lived in a wetter habitat with numerous watercourses and marshes in the open long grass savannah in Garamba National Park in the Democratic Republic of Congo [67] or in the Shambe area in the Sudan in seasonally flooded grasslands, wooded savannah and swamps [68]. As the genetic divergence indicates a separation time over a million years between the northern and southern white rhinos [27], ecological differences could have affected certain parameters of their vocalizations. Lower frequencies of pure tones attenuate (lose the signal intensity) more rapidly in grassland than in marsh habitat due to the ground effect [69]. A longer call duration, a lower minimum frequency of the first distribution of frequency amplitude and less noise in an inhalation of northern white rhinoceros pant calls might possibly serve to increase the chance of signal detection in a wetter habitat and therefore be favoured in selection.

### Individual Signatures in Pant Calls

We showed that pant calls of both species can be classified to correct individuals with a high success, however, pant calls were also highly variable within individuals. The total information capacity of the pant calls of adult northern and southern white rhinos recorded in isolation was modest (3.19 and 3.15 bits, respectively) and would allow for a discrimination of approximately nine individuals of both species based on the call parameters used [52]. Budde and Klump [6] tested the harmonic begging calls of captive black rhinos for individual differences, but found only low inter-individual variation. White rhinos are the most social of all the rhinoceros species [9]–[14] and in evolution, their sociability might select for an increased need to discriminate individuals. In sciurid rodents, species living in larger social groups have more signature information in their alarm calls than species living in smaller groups, which indicates a strong evolutionary link between the social group size and the vocal individuality [49]. The most common group size of females, subadults and juveniles of southern white rhinos is 2–3 individuals although long-term associations of up to six animals have also been observed [10], [15]. The signature information of the pant calls would therefore be sufficient for vocal recognition between the members of the groups, between territorial and subordinate males or neighbouring territorial males. A similar information capacity, which we found in pant, was described in marmot alarm calls (3.37 bits) [70] and the playback experiments revealed that the marmots were indeed able to extract this information as they discriminated between the alarm calls from different individuals [19].

## Conclusions

The variation between individuals, species, contexts and age classes which we found in the contact pant call of northern and southern white rhinos represents the first information reported concerning any rhinoceros call. Playback studies are now needed to investigate if the rhinos are able to extract this information. Understanding the communication of white rhinos is critical as the lack of social relationships with conspecifics and arising communication problems amongst captive rhinos might be one of the contributing factors to their low reproduction. This is crucial particularly for the northern white rhinoceros, which ranks among the most endangered mammals in the world. A knowledge of chemical communication and manipulation of chemosignals has been very successfully implemented, for instance, in the captive breeding programme of giant pandas [71]. We emphasize the need for further research on rhinoceros communication and believe that manipulated communication signals might potentially be used in rhinoceros conservation and management and might help to encourage breeding in captive rhinos.

## Supporting Information

Table S1 Description of acoustic variables entered into the discriminant function analyses.
(DOCX)

Table S2 Descriptive statistics of acoustic variables entered into the discriminant function analyses.
(DOCX)

## References

[1] McComb K, Reby D, Baker L, Moss C, Sayialel S (2003) Long-distance communication of acoustic cues to social identity in African elephants. Anim Behav 65: 317–329.

[2] Soltis J, Leong K, Savage A (2005) African elephant vocal communication II: rumble variation reflects the individual identity and emotional state of callers. Anim Behav 70: 589–599.

[3] Stoeger AS, Heilmann G, Zeppelzauer M, Ganswindt A, Hensman S, et al (2012) Visualizing Sound Emission of Elephant Vocalizations: Evidence for Two Rumble Production Types. PLoS One 7: e48907. 23155427 10.1371/journal.pone.0048907 PMC3498347

[4] Baskin Y (1991) Rhino Biology. Keeping Tabs on an Endangered Species. Science 252: 1256–1257. 17842948 10.1126/science.252.5010.1256

[5] von Muggenthaler EK, Stoughton JW, Daniel JC Jr (1993) Infrasound from the Rhinocerotidae. International Rhino Conference in San Diego, California, 9–11 May 1991.

[6] Budde C, Klump GM (2003) Vocal repertoire of the black rhino *Diceros bicornis* ssp. and possibilities of individual identification. Mamm Biol 68: 42–47.

[7] von Muggenthaler EK, Reinhart P, Lympany B, Craft RB (2003) Songlike vocalizations from the Sumatran rhinoceros (*Dicerorhinus sumatrensis*). Acoust Res Lett Online 4: 83–88.

[8] Policht R, Tomášová K, Holečková D, Frynta D (2008) The vocal repertoire in northern white rhinoceros (*Ceratotherium simum cottoni*) as recorded in the last surviving herd. Bioacoustics 18: 69–96.

[9] Goddard J (1967) Home range, behaviour and recruitment rates of two black rhinoceros populations. East African Wildlife Journal 5: 133–150.

[10] Owen-Smith RN (1973) The behavioural ecology of the white rhinoceros. Ph.D. thesis. Madison: Wisconsin University. 785 p.

[11] Owen-Smith RN (1975) The social ethology of the white rhinoceros *Ceratotherium simum* (Burchell 1817*). Zeitschrift für Tierpsychologie 38: 337–384.

[12] Laurie A (1982) Behavioural ecology of the Greater one-horned rhinoceros (*Rhinoceros unicornis*). J Zool 196: 307–341.

[13] Penny M (1987) Rhinos: an endangered species. Kent: Christopher Helm Publishers Limited. 116 p.

[14] van Gyseghem R (1984) Observations on the ecology and behaviour of the northern white rhinoceros (*Ceratotherium simum cottoni*). Zeitschrift für Säugetierkunde 49: 348–358.

[15] Shrader AM, Owen-Smith N (2002) The role of companionship in the dispersal of white rhinos (*Ceratotherium simum*). Behav Ecol Sociobiol 52: 255–261.

[16] Hammerschmidt K, Fischer J (1998) Maternal discrimination of offspring vocalizations in Barbary macaques (*Macaca sylvanus*). Primates 39: 231–236.

[17] Illmann G, Schrader L, Špinka M, Šustr P (2002) Acoustical mother-offspring recognition in pigs (*Sus scrofa domestica*). Behaviour 139: 487–505.

[18] Charrier I, Mathevon N, Jouventin P (2001) Mother’s voice recognition by seal pups. Nature 412: 873. 10.1038/35091136 11528465

[19] Blumstein DT, Daniel JC (2004) Yellow-bellied marmots discriminate between the alarm calls of individuals and are more responsive to calls from juveniles. Anim Behav 68: 1257–1265.

[20] Schuchmann M, Puechmaille SJ, Siemers BM (2012) Horseshoe Bats Recognise the Sex of Conspecifics from Their Echolocation Calls. Acta Chiropt 14: 161–166.

[21] Frommolt KH, Goltsman ME, MacDonald DW (2003) Barking foxes, *Alopex lagopus*: field experiments in individual recognition in a territorial mammal. Anim Behav 65: 509–518.

[22] Lemasson A, Boutin A, Boivin S, Blois-Heulin C, Hausberger M (2009) Horse (*Equus caballus*) whinnies: a source of social information. Anim Cogn 12: 693–704. 19449192 10.1007/s10071-009-0229-9

[23] Fischer J (2004) Emergence of individual recognition in young macaques. Anim Behav 67: 655–661.

[24] Proops L, McComb K, Reby D (2009) Cross-modal individual recognition in domestic horses (*Equus caballus*). Proc Natl Acad Sci 106: 947–951. 19075246 10.1073/pnas.0809127105 PMC2630083

[25] Insley SJ, Holt MM (2012) Do male northern elephant seals recognize individuals or merely relative dominance rank? J Acoust Soc Am 131: 1. 22280727 10.1121/1.3665259

[26] Ryan M, Kime N (2003) Selection on Long-Distance Acoustic Signals. In: Simmons A, Fay R, Popper A, editors. Springer Handbook of Auditory Research; Acoustic Communication. Berlin: Springer Verlag. 225–274.

[27] Groves CP, Fernando P, Robovský J (2010) The Sixth Rhino: A Taxonomic Re-Assessment of the Critically Endangered Northern White Rhinoceros. PLoS One 5: e9703. 20383328 10.1371/journal.pone.0009703 PMC2850923

[28] Rookmaaker K (2011) A review of black rhino systematics proposed in Ungulate Taxonomy by Groves and Grubb (2011) and its implications for rhino conservation. Pachyderm 50: 72–76.

[29] Rookmaaker K (2014) Nile rhinoceros species account. Available: http://www.rhinoresourcecenter.com/species/nile-rhino/. Accessed 12 March 2014.

[30] Heller E (1913) The white rhinoceros. Smithsonian Miscellaneous Collection 61: 1–56.

[31] Estes RD (1991) The behavior guide to African mammals, including hoofed mammals, carnivores, primates. Berkeley, Los Angeles, London: The University of California Press. 610 p.

[32] Wiley RH, Richards DG (1978) Physical Constraints on Acoustic Communication in the Atmosphere: Implications for the Evolution of Animal Vocalizations. Behav Ecol Sociobiol 3: 69–94.

[33] Charlton BD, Reby D, McComb K (2007) Female red deer prefer the roars of larger males. Biol Lett 3: 382–385. 17550876 10.1098/rsbl.2007.0244 PMC2390678

[34] Terry AMR, Peake TM, McGregor PK (2005) The role of vocal individuality in conservation. Front Zool 2: 10. 15960848 10.1186/1742-9994-2-10 PMC1183234

[35] Knight M (2011) African Rhino Specialist Group report. Pachyderm 49: 6–15.

[36] Hermes R, Hildebrandt TB, Blottner S, Walzer C, Silinski S, et al (2005) Reproductive soundness of captive southern and northern white rhinoceros (*Ceratotherium simum simum, C.s. cotttoni*): evaluation of male genital tract morphology and semen quality before and after cryopreservation. Theriogenology 63: 219–238. 15589286 10.1016/j.theriogenology.2004.04.007

[37] Hermes R, Hildebrandt TB, Walzer C, Göritz F, Patton ML, et al (2006) The effect of long non-reproductive periods on the genital health in captive female white rhinoceros (*Ceratotherium simum simum, C.s. cottoni*). Theriogenology 65: 1492–1515. 16213012 10.1016/j.theriogenology.2005.09.002

[38] Kuneš M, Bičík V (2002) Social and sexual behaviour in captive breeding groups of white rhinoceros. Acta Univ Palacki Olomuc, Fac Rer Nat, Biol 39–40: 81–99.

[39] Swaisgood RR, Dickman DM, White AM (2006) A captive population in crisis: Testing hypotheses for reproductive failure in captive-born southern white rhinoceros females. Biol Conserv 129: 468–476.

[40] Metrione LC, Penfold LM, Waring GH (2007) Social and spatial relationships in captive southern white rhinoceros (*Ceratotherium simum simum*). Zoo Biol 26: 487–502. 19360596 10.1002/zoo.20143

[41] Cinková I, Bičík V (2013) Social and reproductive behaviour of critically endangered northern white rhinoceros in a zoological garden. Mamm Biol 78: 50–54.

[42] Beecher MD (1989) Signalling systems for individual recognition: an information theory approach. Anim Behav 38: 248–261.

[43] Mundry R, Sommer C (2007) Discriminant function analysis with nonindependent data: consequences and an alternative. Anim Behav 74: 965–976.

[44] Venables WN, Ripley BD (2002) Modern Applied Statistics with S. New York: Springer. 497 p.

[45] Arnold BD, Wilkinson GS (2011) Individual specific contact calls of pallid bats (*Antrozous pallidus*) attract conspecifics at roosting sites. Behav Ecol Sociobiol 65: 1581–1593.

[46] Carter GG, Logsdon R, Arnold BD, Menchaca A, Medellin RA (2012) Adult Vampire Bats Produce Contact Calls When Isolated: Acoustic Variation by Species, Population, Colony and Individual. PLoS One 7: e38791. 22719947 10.1371/journal.pone.0038791 PMC3375292

[47] O’Connor BP (2000) SPSS and SAS Programs for Determining the Number of Components Using Parallel Analysis and Velicer’s MAP Test. Behav Res Methods Instrum Comput 32: 396–402. 11029811 10.3758/bf03200807

[48] Sebe F, Duboscq J, Aubin T, Ligout S, Poindron P (2010) Early vocal recognition of mother by lambs: contribution of low- and high-frequency vocalizations. Anim Behav 79: 1055–1066.

[49] Pollard KA, Blumstein DT (2011) Social Group Size Predicts the Evolution of Individuality. Curr Biol 21: 413–417. 21333537 10.1016/j.cub.2011.01.051

[50] Blumstein DT, McClain DR, de Jesus C, Alarcón-Nieto G (2012) Breeding bird density does not drive vocal individuality. Curr Zool 58: 765–772.

[51] Bouchet H, Blois-Heulin C, Lemasson A (2013) Social complexity parallels vocal complexity: a comparison of three non-human primate species. Front Psychol 4: 390. 23847565 10.3389/fpsyg.2013.00390 PMC3705190

[52] Beecher MD (1982) Signature Systems and Kin Recognition. Am Zool 22: 477–490.

[53] Charlton BD, Zhihe Z, Snyder RJ (2009) Vocal cues to identity and relatedness in giant pandas (*Ailuropoda melanoleuca*). J Acoust Soc Am 126: 2721–2732. 19894848 10.1121/1.3224720

[54] Crockford C, Herbinger I, Vigilant L, Boesch C (2004) Wild Chimpanzees Produce Group-Specific Calls: a Case for Vocal Learning? Ethology 110: 221–243.

[55] Ey E, Pfefferle D, Fischer J (2007) Do age- and sex-related variations reliably reflect body size in non-human primate vocalizations? A review. Primates 48: 253–267. 17226064 10.1007/s10329-006-0033-y

[56] Aubin T, Jouventin P (2002) Localisation of an acoustic signal in a noisy environment: the display call of the king penguin *Aptenodytes patagonicus* . J Exp Biol 205: 3793–3798. 12432003 10.1242/jeb.205.24.3793

[57] Rubenstein DI, Hack MA (1992) Hoarse signals: the sounds and scents of fury. Evol Ecol 6: 254–260.

[58] Endler JA (1992) Signals, signal conditions, and the direction of evolution. Am Nat 139: S125–S153.

[59] Policht R, Karadžos A, Frynta D (2011) Comparative Analysis of Long-Range Calls in Equid Stallions (*Equidae*): Are Acoustic Parameters Related to Social Organization? Afr Zool 46: 18–26.

[60] Mikulica V (1991) Social behaviour in two captive groups of white rhinoceros (*Ceratotherium simum simum* and *Ceratotherium simum cottoni*). Zoologische Garten N. F. 61: 365–385.

[61] Cinková I, Ganslosser U, Kretzschmar P (2009) Social behaviour of southern white rhinoceros (*Ceratotherium simum simum*) in game reserves in South Africa. 7^th^ International Conference on Behaviour, Physiology and Genetics of Wildlife in Berlin, Germany, 21–24 September 2009.

[62] Boughman JW (2002) How sensory drive can promote speciation. Trends Ecol Evol 17: 571–577.

[63] Milliken T, Shaw J (2012) The South Africa – Viet Nam Rhino Horn Trade Nexus: A deadly combination of institutional lapses, corrupt wildlife industry professionals and Asian crime syndicates. Johannesburg: TRAFFIC. 173 p.

[64] Pienaar DJ, Bothma J du P, Theron GK (1992) Landscape preference of the white rhinoceros in the southern Kruger National Park. Koedoe 35: 1–7.

[65] Pienaar DJ, Bothma J du P, Theron GK (1993) Landscape preference of the white rhinoceros in central and northern Kruger National Park. Koedoe 36: 79–85.

[66] Emslie R (2011) The IUCN Red List of Threatened Species. *Ceratotherium simum* ssp. *cottoni* Available: http://www.iucnredlist.org/details/4183/0. Accessed 12 March 2014.

[67] Hillman-Smith K (1987) Northern white rhinos in Garamba National Park. Pachyderm 9: 19–22.

[68] Hillman-Smith K (1982) Sudan, white rhino – Proposed Shambe National Park. WWF Yearbook 1982: 273–276.

[69] Cosens SE, Falls JB (1984) A comparison of sound propagation and song frequency in temperate marsh and grassland habitats. Behav Ecol Sociobiol 15: 161–170.

[70] Blumstein DT, Munos O (2005) Individual, age and sex-specific information is contained in yellow-bellied marmot alarm calls. Anim Behav 69: 353–361.

[71] Swaisgood RR, Lindburg D, White AM, Zhang H, Zhou X (2004) Chemical communication in giant pandas: experimentation and application. In: Lindburg D, Baragona K, editors. Giant Pandas: Biology and Conservation. Berkeley: University of California Press. 106–120.

