# Supplementary material for: Contact Calls of the Northern and Southern White Rhinoceros Allow for Individual and Species Identification
Source: PLoS One. 2014 Jun 5;9(6):e98475. doi: 10.1371/journal.pone.0098475 (PMC4047034; doi:10.1371/journal.pone.0098475)
Supplement: Table S1 — Description of acoustic variables entered into the discriminant function analyses. (DOCX) [file pone.0098475.s001.docx]

**Table S1.** Description of acoustic variables entered into the discriminant function analyses.

| **Acoustic variable^1^** | **Description** | **Software** | **Inclusion of parameter into DFAs^2^** | | |  |
| --- | --- | --- | --- | --- | --- | --- |
|  |  |  | **DFA 1,2** | **DFA 3** | **DFA 4** |  |
| Call duration | Duration of the call [sec] | Avisoft |  | * |  |  |
| No. of elements | Number of elements in call | Avisoft |  | * |  |  |
| I: max element length | Duration of the longest inhalation in call [sec] | Avisoft | * |  |  |  |
| E: max element length | Duration of the longest exhalation in call [sec] | Avisoft | * | * | * |  |
| I: min element length | Duration of the shortest inhalation in call [sec] | Avisoft | * | * |  |  |
| I: no. in cat. 1,2 – percentage | Percentage of inhalations in duration from 0.0–0.4 sec (= in categories 1,2) from the total number of inhalations in call | Avisoft |  | * | * |  |
| E: no. in cat.1,2 | No. of exhalations in call in duration from 0.0–0.4 sec (= in categories 1,2) | Avisoft | * |  |  |  |
| E: duration | Duration of exhalation [sec] | Avisoft |  | * | * |  |
| Order longest inhalation | Order of the longest inhalation in call | Avisoft |  | * | * |  |
| I: peak frequency (max) | Frequency of maximum amplitude of the spectrum [Hz] | Avisoft | * | * |  |  |
| I: entropy (mean) | Ratio of geometric mean to the arithmetic mean of spectrum; allows to quantify the randomness of the sounds | Avisoft | * |  |  |  |
| I: entropy (std) | Ratio of geometric mean to the arithmetic mean of spectrum; allows to quantify the randomness of the sounds | Avisoft |  | * |  |  |
| I: hnr (std) | Ratio of harmonic to nonharmonic energy | Avisoft |  | * | * |  |
| I: ampratio2 | Amplitude ratio between 1^st^ and 3^rd^ dominant frequency band | LMA |  | * |  |  |
| I: ampratio3 | Amplitude ratio between 2^nd^ and 3^rd^ dominant frequency band | LMA | * |  |  |  |
| I: q3min | Minimum frequency of the 3^rd^ distribution of frequency amplitude [Hz] | LMA |  | * |  |  |
| I: ranmean | Mean frequency range [Hz] | LMA |  | * | * |  |
| I: fp1amax | Maximum amplitude of the 1^st^ global frequency peak [relative amplitude] | LMA |  | * |  |  |
| I: df3mean | Mean frequency of the 3^rd^ dominant frequency band [Hz] | LMA | * |  |  |  |
| I: pfmin | Minimum peak frequency [Hz] | LMA |  | * | * |  |
| E: fp1amean | Mean amplitude of the 1^st^ global frequency peak [relative amplitude] | LMA |  | * | * |  |
| E: pftotmax | Frequency of the total maximum amplitude [Hz] | LMA | * |  |  |  |
| E: pftrmean | Mean deviation between peak frequency and linear trend [Hz] | LMA |  | * |  |  |

**^1^**I = parameter measured in inhalation; E = parameter measured in exhalation; (max) = parameter measured at the location of maximum amplitude; (mean) = parameter derived from the mean spectrum of entire element; (std) parameter is computed for all spectra between the start and the end of each element and the relative standard deviation of these parameters is calculated, this parameter can be used for quantifying frequency or amplitude modulation.

**^2^**An asterisk shows if particular variable was included in DFAs: DFA 1 = DFA conducted with calls of all the northern white rhinos recorded in various contexts, DFA 2 = DFA conducted with calls of adult northern white rhinos recorded in isolation, DFA 3 = DFA conducted with calls of all the southern white rhinos recorded in various contexts, DFA 4 = DFA conducted with calls of adult southern white rhinos recorded in isolation.
