# Supplementary material for: Contact Calls of the Northern and Southern White Rhinoceros Allow for Individual and Species Identification
Source: PLoS One. 2014 Jun 5;9(6):e98475. doi: 10.1371/journal.pone.0098475 (PMC4047034; doi:10.1371/journal.pone.0098475)
Supplement: Table S2 — Descriptive statistics of acoustic variables entered into the discriminant function analyses. (DOCX) [file pone.0098475.s002.docx]

**Table S2.** Descriptive statistics of acoustic variables entered into the discriminant function analyses.

| **Acoustic variable** | **Northern white rhinos** | | **Southern white rhinos** | |
| --- | --- | --- | --- | --- |
|  | Mean | sd | Mean | sd |
| Call duration [sec] | 6.1 | 1.76 | 3.9 | 0.95 |
| No. of elements in call | 14.1 | 4.67 | 9.2 | 2.64 |
| I: max element length [sec] | 1.0 | 0.25 | 0.6 | 0.23 |
| E: max element length [sec] | 0.4 | 0.17 | 0.4 | 0.13 |
| I: min element length [sec] | 0.3 | 0.11 | 0.2 | 0.07 |
| I: no. in cat. 1,2 – percentage | 0.6 | 0.19 | 0.7 | 0.2 |
| E: no. in cat. 1,2 | 6.2 | 2.49 | 3.3 | 1.65 |
| E: duration [sec] | 0.3 | 0.09 | 0.3 | 0.08 |
| Order longest inhalation | 3.6 | 3.73 | 3.5 | 2.51 |
| I: peak frequency (max) [Hz] | 967.3 | 695.79 | 1345.5 | 439.14 |
| I: entropy (mean) | 0.3 | 0.06 | 0.4 | 0.05 |
| I: entropy (std) | 0.1 | 0.05 | 0.1 | 0.04 |
| I: hnr (std) | 0.1 | 0.04 | 0.1 | 0.03 |
| I: ampratio2 | 1.5 | 0.72 | 1.4 | 0.67 |
| I: ampratio3 | 1.4 | 0.41 | 1.3 | 0.31 |
| I: q3min [Hz] | 1542.0 | 658.91 | 2346.7 | 753.57 |
| I: ranmean [Hz] | 4793.6 | 1609.58 | 4504.6 | 1362.65 |
| I: fp1amax | 305.9 | 293.22 | 496.5 | 333.56 |
| I: df3mean [Hz] | 2578.1 | 853.86 | 2532.5 | 736.07 |
| I: pfmin [Hz] | 84.5 | 96.64 | 222.9 | 194.17 |
| E: fp1amean | 92.8 | 123.32 | 77.5 | 68.72 |
| E: pftotmax [Hz] | 326.6 | 449.38 | 930.9 | 675.68 |
| E: pftrmean [Hz] | 895.1 | 620.55 | 1438.6 | 624.27 |

Descriptive statistics were calculated as averages of mean values/individual and included pant calls recorded from all the animals in various social contexts. I = parameter measured in inhalation, E = parameter measured in exhalation.
